# Cell Recruitment and Cytokines in Skin Mice Sensitized with the Vaccine Adjuvants: Saponin, Incomplete Freund’s Adjuvant, and Monophosphoryl Lipid A

**DOI:** 10.1371/journal.pone.0040745

**Published:** 2012-07-19

**Authors:** Juliana Vitoriano-Souza, Nádia das Dores Moreira, Andréa Teixeira-Carvalho, Cláudia Martins Carneiro, Fernando Augusto Mathias Siqueira, Paula Melo de Abreu Vieira, Rodolfo Cordeiro Giunchetti, Sandra Aparecida de Lima Moura, Ricardo Toshio Fujiwara, Maria Norma Melo, Alexandre Barbosa Reis

**Affiliations:** 1 Departamento de Parasitologia, Instituto de Ciências Biológicas, Universidade Federal de Minas Gerais, Belo Horizonte, Minas Gerais, Brasil; 2 Laboratório de Imunopatologia, Núcleo de Pesquisas em Ciências Biológicas (NUPEB), Universidade Federal de Ouro Preto (UFOP), Campus Universitário Morro do Cruzeiro, Ouro Preto, Minas Gerais, Brazil; 3 Laboratório de Biomarcadores de Diagnóstico e Monitoração, Centro de Pesquisas René Rachou, Fundação Oswaldo Cruz, 30190-002, Belo Horizonte, Minas Gerais, Brazil; 4 Departamento de Análises Clínicas, Escola de Farmácia, Universidade Federal de Ouro Preto, Ouro Preto, Minas Gerais, Brazil; Statens Serum Institute, Denmark

## Abstract

Vaccine adjuvants are substances associated with antigens that are fundamental to the formation of an intense, durable, and fast immune response. In this context, the use of vaccine adjuvants to generate an effective cellular immune response is crucial for the design and development of vaccines against visceral leishmaniasis. The objective of this study was to evaluate innate inflammatory response induced by the vaccine adjuvants saponin (SAP), incomplete Freund’s adjuvant (IFA), and monophosphoryl lipid A (MPL). After a single dose of adjuvant was injected into the skin of mice, we analyzed inflammatory reaction, selective cell migration, and cytokine production at the injection site, and inflammatory cell influx in the peripheral blood. We found that all vaccine adjuvants were able to promote cell recruitment to the site without tissue damage. In addition, they induced selective migration of neutrophils, macrophages, and lymphocytes. The influx of neutrophils was notable at 12 h in all groups, but at other time points it was most evident after inoculation with SAP. With regard to cytokines, the SAP led to production of interleukin (IL)-2, IL-6, and IL-4. IFA promoted production of tumor necrosis factor (TNF)-α, interferon (IFN)-γ, IL-6, IL-17, IL-4, and IL-10. We also observed that MPL induced high production of IL-2, TNF-α, and IFN-γ, in addition to IL-6, IL-17, and IL-10. In peripheral blood, values of certain cell populations in the local response changed after stimulation. Our data demonstrate that the three vaccine adjuvants stimulate the early events of innate immune response at the injection site, suggesting their ability to increase the immunogenicity of co-administered antigens. Moreover, this work provides relevant information about elements of innate and acquired immune response induced by vaccine adjuvants administered alone.

## Introduction

The main goal of vaccination is the development of a potent memory response by T cells against specific pathogens, an event that seems to occur in the first week after immunization or infection [1]. Thus, the research on efficient vaccination focuses not only on the delivery method and administration route, but also on the composition and safety of vaccines [2], [3]. In this context, vaccine adjuvants are very important additives. They enhance the immunogenicity of an antigen by aiding in the formation of an intense and prolonged immune response in the presence of smaller amounts of antigen, decreasing costs and reducing problems such as competition between antigens [4], [5]. Many adjuvants were found empirically, and progress to understand their mechanism of action has been slow, which partly explains why the number of adjuvants approved for human use is still low [6]. For a long time, colloidal aluminum salts (alum) were the only approved adjuvants, but more recently squalene emulsions (MF59) and monophosphoryl lipid A (MPL) have been licensed for usage in Europe [7].

Considering the scarcity of information regarding the mechanisms of immune action triggered by vaccine adjuvants in preclinical studies, we selected classical and modern adjuvants, including saponin (SAP), incomplete Freund’s adjuvant (IFA), and MPL, for study. It is noteworthy that there is a remarkable difference between administration of antigen alone, which results in little or no response, and administration of antigen plus adjuvant, which results in priming and the ability to induce an inflammatory response. In this sense, inflammatory cytokines produced by macrophages or innate immune cells at the injection site may be essential communicators of adjuvant activity [6]–[8].

Regarding vaccine adjuvants, saponins induce an intense immune response type 1 (CTL-mediated cytotoxicity by T lymphocytes and IgG2a) and a concomitant response type 2 [9]. Saponins are natural glycosides of steroids or triterpenes obtained from the bark of *Quillaja saponaria*. Also, saponins are ideal for use in vaccine trials against *Leishmania* spp. [10], since they have low-cost, simple formulations and are generally considered safe for veterinary use [11]. Another classical adjuvant is IFA [12], an aqueous solution that contains mineral oil and an emulsifying agent. The most commonly used emulsifying agent in IFA is mannide monooleate, which causes the dispersal of small droplets of the oil. Further modified IFA systems (water-in-oil emulsions) were then developed, such as Montanide ISA 51, which contains mineral oil and mannide monooleate as a surfactant. It seems to generate a quality and intensity of immunogenicity similar to aluminum hydroxide but presents several undesirable side effects, including granuloma formation, local pain, tenderness, and erythema [13].

More recently, adjuvant systems have used MPL in combination with various adjuvants such as the following: AS02, MPL and a purified fraction of QS-21 saponin; AS03, emulsion containing α- tocopherol and squalene; and AS04, an emulsion of MPL plus aluminium salt [10]. It is well known that MPL is a detoxified (chemically modified) form of the endotoxin lipopolysaccharide (LPS) from *Salmonella minnesota*. Similar to LPS, it exerts its action through toll-like receptor (TLR) 4. TLR agonists are potent activators of the innate immune response through activating dendritic cell maturation and inflammatory cytokine secretion by innate immune cells, and they consequently induce an adaptive immunity when co-administered with a foreign antigen [14].

Here, we have investigated the particular immune response to these three adjuvants through analysis of inflammatory reaction, selective cell migration, cytokine production, and inflammatory cell influx in the peripheral blood after injection of a single dose in the skin of mice. We hypothesized that these adjuvants induce an innate immune response at injection sites and thus create an immune microenvironment with the necessary elements to facilitate vaccine-triggered adaptive immunity. Herein we show that the three adjuvants studied were able to induce inflammation with different cells migrating after sensitization, an important event for the initiation of the immune response.

## Materials and Methods

### Animals and Immunization Protocol

Male outbred Swiss albino mice (8–10 weeks old) were purchased from the Centro de Ciência Animal (CCA/UFOP) and kept in ventilated racks with food and water *ad libitum* throughout the study. The protocol for the animal experiments was approved by the Câmara de Experimentação Animal do Comitê de Ética (CEP No. 008/2009), Departamento de Ciências Biológicas, Universidade Federal de Ouro Preto (UFOP).

Mice were inoculated intradermally in the back with a single dose of vaccine adjuvant and evaluated at several time points afterward (1, 12, 24, 48, 96, 168, and 336 h). A visible raised cutaneous swelling was regarded as evidence of successful intradermal administration. To evaluate the effects caused by vaccine adjuvant and adjuvant-specific responses, the animals were divided into four experimental groups (n = 5 animals/group/time): SAP group, inoculated with 100 µg/dose of saponin (Sigma Chemical Co., St. Louis, MO); IFA group, inoculated with 50 µL/dose of IFA (Sigma Chemical Co.); MPL group, inoculated with 50 µg/dose of MPL-SE® adjuvant (stable emulsion, Corixa, Hamilton, MT); and control group, inoculated with 50 µL of 0.9% sterile saline. The doses used for each adjuvant were inoculated in animals in 50 µL volumes. Mice were euthanized at the indicated time points after injection, the blood was collected for immunophenotyping of peripheral blood cells (24 and 48 h). Skin samples were collected for histological analysis and cytokine assessment. All experiments were performed using groups of five animals/evaluation time in two independent experiments.

### Histological Examination

Skin biopsies from the inoculation sites were fixed in 10% formalin, processed, embedded in paraffin, cut by microtome into 5 µm sections, and mounted on slides. Sections were stained with hematoxylin and eosin for quantification of cellular infiltration and differential counts of inflammatory cells. For the quantification of cellular infiltration, the slides were photographed and analyzed by using image analysis by morphometry. The procedure used was based on the count of inflammatory cells present in the skin through the acquisition of 20 random images (total area covered equal to 1.5×10^6^ µm^2^). Images viewed by 40× objective were digitized through a microscope Leica DM5000B, which has a miniature camera attached to it, and the program Leica Application Suite (version 2.4.0 R1, Leica Microsystems Ltd., Heerbrugg, Switzerland). The image analyses were performed using the Leica Qwin V3 (Leica Microsystems Ltd.), counting all cell nuclei.

### Cytometric Bead Array

Skin was homogenized using a tissue homogenizer (Homo mix) in 1 mL of specific buffer for cytokine extraction (0.4 M NaCl, 0.05% Tween 20, 0.5% bovine serum albumin, 0.1 mM phenyl methyl sulfonyl hydrofluoric, 0.1 mM benzethonium chloride, 10 mM ethylenediamine tetra-acetic acid [EDTA], and 20 KI of aprotinin). The homogenates were centrifuged at 10,000×*g* for 10 min at 4°C, and supernatants were stored at −70°C before analysis. Cytokine levels were measured by Cytometric Bead Array (BD Biosciences) according to the manufacturer’s recommendations. The cytokines evaluated were interleukin (IL)-2, IL-4, IL-6, IL-10, IL-17A, interferon (IFN)-γ, and tumor necrosis factor (TNF)-α. Standard curves for each cytokine were plotted and the concentrations of each test sample in picograms per milliliter (pg/mL) were calculated using the FCAP software array v.1.0.2 (BD Biosciences).

### Blood Sample Collection

Fifty microliters of peripheral blood was collected from the retro-orbital complex of each mouse and transferred to tubes containing EDTA (Sigma Chemical Co) as anticoagulant. The absolute count of leukocytes in each sample was obtained using an Auto Hematology Analyzer (Mindray BC-2800Vet, Hamburg, Germany). The differential cell count was performed with Giemsa-stained smears to determine the absolute number of neutrophils, eosinophils, lymphocytes, and monocytes, using an optical microscopy immersion objective and counting 100 leukocytes/slide.

### Immunophenotyping of Blood Cells by Flow Cytometry

The immunophenotyping of blood cells at 24 and 48 h was performed by flow cytometry. The markers used were monoclonal antibodies against CD14 (FITC Rat anti-Mouse CD14, clone Sa2-8, eBioscience), CD3 (PE Hamster anti-Mouse CD3, clone 145-2C11, Biolegend), CD4 (PercP-Cy™ 5.5 Rat anti-Mouse CD4, clone RM4-5, BD Pharmingen™), CD8 (FITC Rat anti-Mouse CD8a, clone 5H10, Catalg), and CD19 (FITC Rat anti-Mouse CD19, clone 6D5, Catalg). The antibodies were added to polystyrene tubes, and 25 µL of peripheral whole blood collected in EDTA was added to each tube. After homogenization in a vortex, the suspensions were incubated for 30 min at room temperature in the dark. After lysis of erythrocytes, the samples were centrifuged at 600×*g* for 7 min at room temperature. The supernatant was discarded and the leucocytes washed with phosphate-buffered saline (pH 7.4), using the same centrifugation conditions described above (600×*g* for 7 min). Afterward, the leukocytes were fixed with 200 µL of FACS FIX solution (10.0 g/L paraformaldehyde, 10.2 g/L sodium cacodylate, and 6.65 g/L sodium chloride, pH 7.2) and stored at 4°C prior to flow cytometric acquisition. Flow cytometric measurements were performed on a FACScalibur® instrument (Becton Dickinson, Moutain View, CA). A total of 15,000 events were acquired for each preparation. The program CELLQuest® (Franklin Lakes, NJ) was used for data acquisition, and the Flow Jo Software (Flow Cytometry Analysis Software 7.6., Tree Star, Inc., Ashland, OR) was used for data analyses. Nonspecific binding was monitored by using fluorochrome-labeled isotypic matched reagents to provide valid negative controls. Autofluorescence was monitored by the use of a negative control in which the cell suspension was incubated in the absence of fluorochrome-labeled monoclonal antibodies, but in the presence of dilution and wash buffers.

### Statistical Analysis

One-way ANOVA followed by Tukey’s test were used to analyze the differences between groups. P values were calculated by PRISM software (GraphPad, San Diego, CA). Pearson correlation was also computed to investigate associations between pro-inflammatory and regulatory cytokine profile into same group.

## Results

### Cellular Infiltrate Kinetics in Mouse Skin after Sensitization with Vaccine Adjuvants

**Figure 1:**
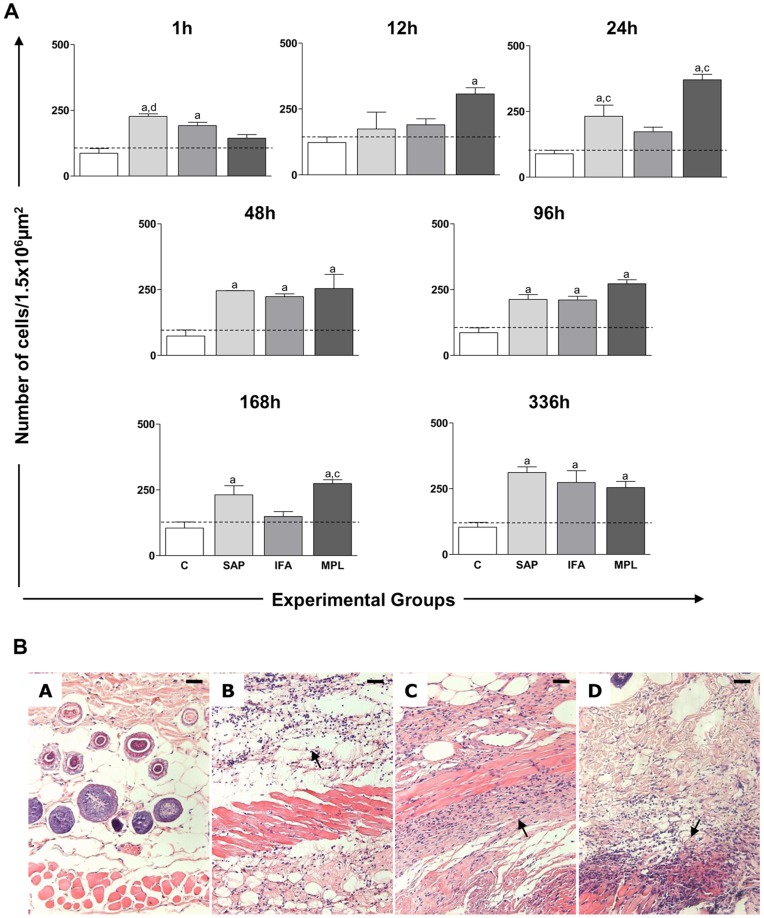
Quantification of the cellular infiltrate in the skin of mice after sensitization with different vaccine adjuvants: saponin (SAP; light gray), incomplete Freund’s adjuvant (IFA; medium gray), and monophosphoryl lipid A (MPL; dark gray) at 1, 12, 24, 48, 96, 168, and 336 h after stimulation. The control group (C; white) was inoculated with 0.9% sterile saline (A). Significant differences (p<0.05) between groups are represented by the letters a, b, c, and d, referring to the C, SAP, IFA, and MPL groups, respectively, and ANOVA following Tukey’s test was employed. The dashed line represents the average number of cell nuclei quantified in histological sections of the skin mouse sensitized with saline. Data presented are the mean±SD from groups of five animals/evaluation time. (B) Representative photomicrographs of the cellular infiltrate at 48 h is shown at the bottom of Figure 1. Skin mice sensitized with saline and the vaccine adjuvants: Saponin (B), IFA (C), MPL (D) at a magnification of 20×; bar  = 100 µm.

We evaluated the kinetics of cell migration induced by the vaccine adjuvants SAP, IFA, and MPL to the site of the injection at 1, 12, 24, 48, 96, 168, and 336 h after sensitization. The results are shown in Figure 1.

At 1 h after inoculation, there was increased cellular infiltrate in the SAP (226.7±17.10) and IFA (191.9±21.50) groups when compared to the control (p<0.05). In the SAP group (226.7±17.10), the increase was also significant when compared to the MPL group (p<0.05). At 12 h we observed an increase (p<0.05) in cellular infiltration in the MPL group (306.9±53.18), but only in comparison to the control. The cellular infiltration increase was detected in the MPL (370.3±35.96) and SAP (231.4±85.45) groups as compared to the control and IFA groups at 24 h. At 48 and 96 h after sensitization, there was an increase in cell migration in SAP (246.1±0.5657; 212.7±31.90), IFA (223.6±20.75; 210.6±29.29), and MPL (253.9±108.3; 272.3±25.81) groups as compared to controls. At 168 h after inoculation, a significant increase (p<0.05) was observed in the cellular infiltrate in the SAP (231.0±68.87) and MPL (274.2±24.61) groups as compared to control. During this time, we also detected an increase in the cell migration in the MPL group in comparison with the IFA group. At the delayed time point (336 h), an increase was observed in the SAP (311.7±36.84), IFA (273.5±77.98), and MPL (254.3±40.60) groups when compared to control (p<0.05).

A typical cellular infiltrate observed in the skin is illustrated in Figure 1B, with photomicrographs showing the cell recruitment within 48 h after sensitization with vaccine adjuvants. The cellular infiltrate was composed mainly of neutrophils, macrophages, and lymphocytes. In the SAP group, the cellular infiltrate was more diffuse throughout the hypodermis layer, mainly in the adipocyte layer. In the IFA group, the cellular infiltrate in the hypodermis was near the muscular layer of the skin. In the MPL group, there were intense inflammatory foci in the muscle layer of the hypodermis. These data show the ability of adjuvants to promote local inflammation, which might be important for the initiation of the innate and acquired immune response. The local inflammatory reaction did not induce macroscopical ulceration (data not shown).

### Differential Leukocyte Counts (Neutrophils, Macrophages, and Lymphocytes) in Inflammatory Mouse Skin Foci after Sensitization with Vaccine Adjuvants

**Figure 2:**
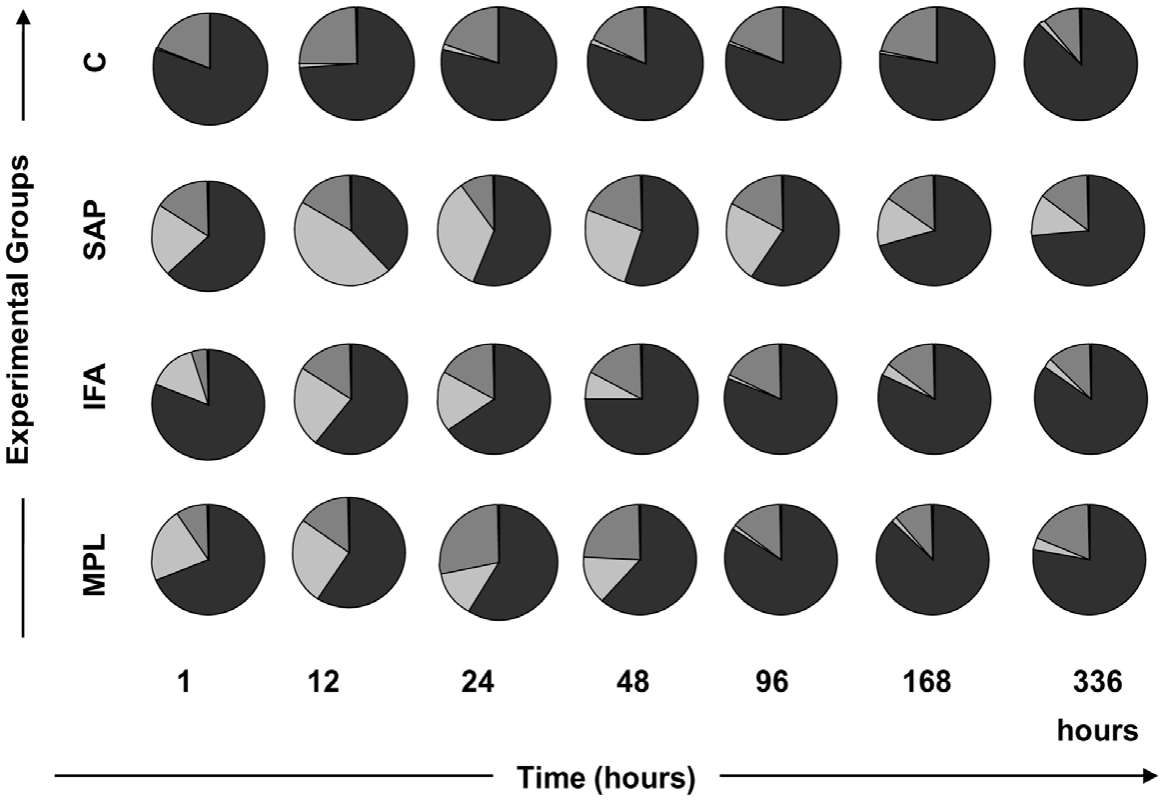
Enumeration of the different cell types (neutrophils, macrophages, and lymphocytes) in the inflammatory focus in mouse skin after sensitization with vaccine adjuvants: saponin (SAP; light gray), incomplete Freund’s adjuvant (IFA; medium gray), and monophosphoryl lipid A (MPL; dark gray) at 1, 12, 24, 48, 96, 168, and 336 h after stimulation. The control group was inoculated with 0.9% sterile saline (C; white). The representative time-lapse graphic demonstrates the major inflammatory cells that migrated to the skin after sensitization: neutrophils (light gray), lymphocytes (medium gray), and macrophages (dark gray). Five mice were used in each group/evaluation time.

After we observed the ability of vaccine adjuvants to induce cellular recruitment in mouse skin, we assessed the differential cell migration in inflammatory foci. To identify the composition of the major immune cells (neutrophils, macrophages, and lymphocytes) recruited to the site after inoculation, we performed a differential cell count in skin sections stained with hematoxylin and eosin, using optical microscopy (Figure 2, pie charts). The results demonstrated that the cellular infiltrate was composed of mononuclear cells (macrophage and lymphocytes) in the control group. This feature may be related to injury caused by inoculation and/or the resident cell population in the skin compartment. In contrast, there was an increased percentage of neutrophils at 12 h with all adjuvants tested. Specifically, SAP induced a higher neutrophil influx to skin at all time points. In contrast, IFA and MPL were great inductors of neutrophils during the early events after sensitization (1–48 h), but after 48 h these adjuvants provoked a reversible neutrophil influx to the inoculation site.

### Proinflammatory and Regulatory Cytokines in Mouse Skin at Different Time Points after Sensitization with Vaccine Adjuvants

**Figure 3:**
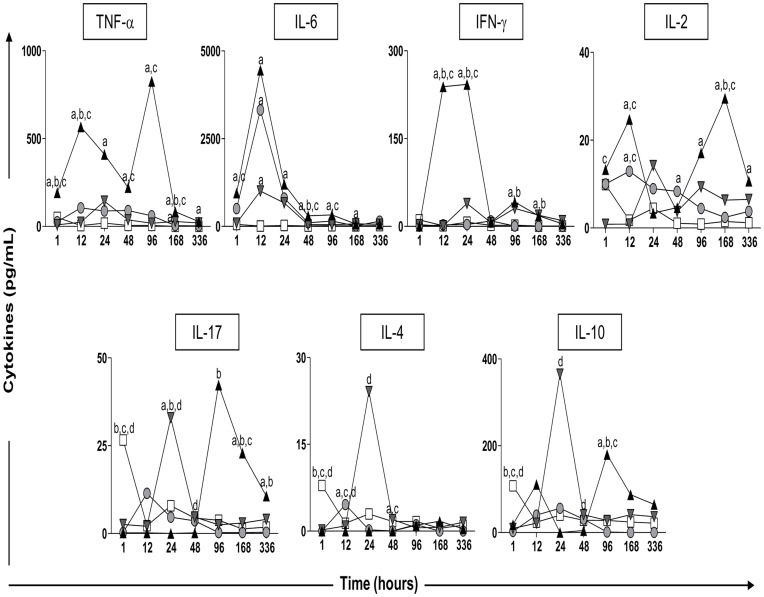
Kinetics of pro-inflammatory and regulatory (TNF-α IL-6, IFN-γ IL-2, IL-17, IL-4, and IL-10) cytokines in the skin of mice after sensitization with vaccine adjuvants: saponin (SAP; gray circle), incomplete Freund’s adjuvant (IFA; gray inverted triangle), and monophosphoryl lipid A (MPL; black triangle) at 1, 12, 24, 48, 96, 168, and 336 h after stimuli. The control group was inoculated with 0.9% sterile saline (C; white square). Significant differences (p<0.05) between groups are represented by the letters a, b, c and d referring to the C, SAP, IFA, and MPL groups, respectively. Five mice were used in each group/evaluation time.

The profile of proinflammatory and regulatory cytokines (TNF-α, IL-6, IFN-γ, IL-2, IL-17, IL-4, and IL-10) was obtained from homogenized skin tissue from the inoculation site. The cytokine levels were measured by CBA kit to identify the cytokines produced by both innate and effector cells as a result of adjuvant stimulation (Figure 3).

Our results showed that SAP induced lower levels of cytokines when compared with the other groups sensitized with adjuvants. We also observed increased levels of IL-2, IL-6, and IL-4 in this group. In the IFA group, we observed induction of TNF-α, IL-6, IFN-γ, IL-17, IL-4, and IL-10 production. Finally, MPL elevated the levels of TNF-α, IL-6, IFN-γ, IL-2, IL-17, and IL-10 at different time points.

**Figure 4:**
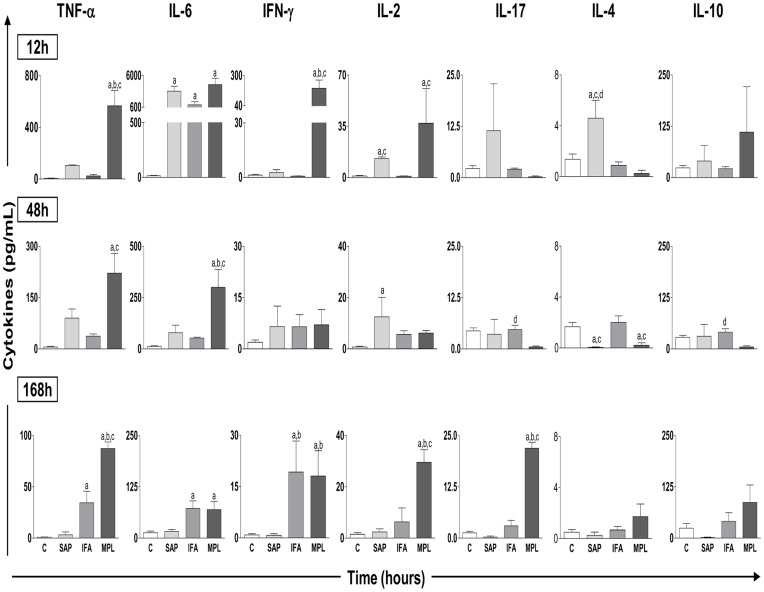
Cytokine pattern in the homogenate of mouse skin after sensitization with vaccine adjuvants saponin (SAP; light gray), incomplete Freund’s adjuvant (IFA; medium gray), and monophosphoryl lipid A (MPL; dark gray) at 12, 48, and 168 h after stimulation. The control group was inoculated with 0.9% sterile saline (C; white). The results are normalized as the ratio between the levels of cytokine (pg/mL) and weight of tissue (mg). Significant differences (p<0.05) between groups are represented by the letters a, b, c and d, referring to the C, SAP, IFA, and MPL groups, respectively, and ANOVA following Tukey’s test was employed. Data presented are the mean±SD from groups of five animals/evaluation time.

Figure 4 shows kinetics at specific time points (12, 48, and 168 h) to highlight major alterations in cytokine production after sensitization with different adjuvants. The results show significant differences in the production profiles of both pro-inflammatory and regulatory cytokines. With regard to TNF-α production at 12 h, levels increased in the MPL group (566.9±235.3) compared to the others groups. At 48 h, the increase in the MPL group (221.5±129.3) remained evident in relation to the control and IFA groups. At 168 h, we observed an increase of TNF-α in the MPL group (87.41±12.53) as compared to the others groups. We also observed an increase in this cytokine levels at 168 h in the IFA group (34.42±25.16) as compared to the control. Regarding the production of IL-6, we observed a peak at 12 h as well as increased levels in the SAP (3316±1459), IFA (1007±917.5), and MPL (4447±2185) groups as compared to the control. At 48 h, a significant increase was noted in the MPL group (300.3±174.7) as compared to the control, SAP, and IFA groups. At 168 h, there was an increase of IL-6 in the IFA (72.39±32.16) and MPL (69.71±40.27) groups as compared to the control. The analysis of IFN-γ showed higher levels of this cytokine at 12 h in the MPL group (190.9±157.7) as compared to the control, SAP, and IFA groups. At 168 h, increased levels of IFN-γ in the IFA (19.23±18.19) and MPL (18.09±14.74) groups were observed as compared to the control and SAP groups. More specifically, the data showed increased production of IL-2 at 12 h in the MPL (37.13±33.39) and SAP (12.93±2.02) groups when compared to the IFA and control groups. At 48 h, there was increased production of IL-2 in the SAP group (12.53±10.68) as compared to the control. At 168 h this increase was again seen in the MPL group (29.55±8.56) as compared to the control, SAP, and IFA groups.

Regarding the cytokine IL-17, there was initially a reduction in the SAP (0.25±0.50), IFA (2.56±1.11), and MPL (0.20±0.44) groups when compared to the control (data not shown). At 48 h, we noticed an increase in IL-17 in the IFA group (4.69±2.38) as compared to the MPL group. At 168 h, increased levels of IL-17 were observed in the MPL group (21.90±2.46) as compared to the control, SAP, and IFA groups.

For IL-4, reduced levels were observed early (at 1 h) in the SAP (0.0±0.0), IFA (0.21±0.32), and MPL (0.0±0.0) groups as compared to the control (data not shown). At 12 h, there were increased levels of IL-4 in the SAP group (4.59±1.99) as compared to the control, IFA, and MPL groups. By 48 h, reduction of this cytokine was observed in the SAP (0.042±0.085) and MPL (0.20±0.44) groups as compared to the control and IFA groups. Reduced levels at 1 h were also observed for IL-10 in the SAP (2.40±4.80), IFA (9.50±2.91), and MPL (18.11±2.52) groups when compared to the control group (data not shown). At 48 h, there was increased IL-10 in the IFA group (40.88±16.34) when compared to the MPL group. At 96 h, IL-10 was increased in the MPL group (179.8±149.9) compared to the control (27.97±12.37), SAP (1.11±2.23), and IFA (27.86±26.34) groups (data not shown).

### Immunophenotyping of Circulating Leukocytes from Peripheral Blood after Sensitization with Different Vaccine Adjuvants

Following evaluation of the compartmentalized immune response, we investigated the systemic response including the hematological profile at 1, 12, 24, 48, 96, 168, and 336 h after sensitization with different vaccine adjuvants (Table 1).

**Table 1:** Hematological profile of peripheral blood from mice after sensitization with vaccine adjuvants: saponin (SAP), incomplete Freund’s adjuvant (IFA), and monophosphoryl lipid A (MPL) at 1, 12, 24, 48, 96, 168, and 336 h after stimulation.

Groups/Adjuvants		Time (h)						
		1	12	24	48	96	168	336
**C**	Total Leukocytes	6900±244.9	5520±2008	6900±3037	6660±1662	6720±440.9	3300±924.7	4080±1134
	Neutrophils	1022±286.5	696.8±352.3	837.0±194.3	396.0±218.3	776.4±211.8	391.2±107.9	816.6±353.3
	Monocytes	69.0±2.4	55.2±20.1	105.0±79.60	79.8±33.6	92.4±33.8	38.2±7.5	58.20±32.97
	Lymphocytes	5810±230.2	4590±1746	5958±2867	6184±1488	5851±805.5	2852±918.7	3196±831.1
**SAP**	Total Leukocytes	3300±793.7 ^a,c^	6000±1897	10350±900 ^c,d^	9000±2728 ^d^	9525±5588^ d^	9075±2765	9750±3969^ a^
	Neutrophils	757.5±430.3	3714±1368 ^a,c^	3740±1679 ^a,c,d^	3018±2044^ a,c,d^	1750±1358	1331±557.2	1661±1378
	Monocytes	39.7±14.9	70.5±17.2	103.5±9.	178.5±48.6^ d^	116.3±65.2	111.0±43.3	145.0±31.2^ a^
	Lymphocytes	2068±721.5^ a,c,d^	2216±638.2	6507±1090	5804±1983	7703±4269^ d^	7603±2439	7898±3749^ a^
**IFA**	Total Leukocytes	6420±962.8	3300±2001	5940±712.5	6360±1635	8160±1332^ d^	11100±2741^ a^	9840±1297^ a^
	Neutrophils	690.0±323.9	1144±696.9	732.0±324.5	827.4±341.3	563.4±86.7	1413±388.5^ a^	1093±330.4
	Monocytes	93.0±69.2	43.5±14.8	103.2±27.9	114.0±76.5	117.0±56.1	135.6±67.2 ^a^	147.0±43.4^ a,d^
	Lymphocytes	5637±666.4	2011±1578^ a^	5093±1439	5419±1506	7480±1357^ d^	9551±2792^ a^	8612±1282^ a^
**MPL**	Total Leukocytes	4800±1358^ a^	5460±1090	6180±1301	4300±458.3	2650±854.4	7150±5123	6900±3050
	Neutrophils	1384±795.4	2428±518.4^ a^	1391±331.7	699.0±366.8	760.5±228.3	987.0±770.5	1179±664.8
	Monocytes	88.8±51.4	73.8±22.5	105.8±97.6	72.0±42.2	65.0±19.4	83.0±48.7	75.7±18.8
	Lymphocytes	3317±573.8^ a,c^	2958±717.3	4704±1319	3009±891.9^ a^	1811±907.4	6080±4394	5623±2603

The control group (C) was inoculated with 0.9% sterile saline. The absolute numbers (mm^3^) of total leukocytes and their subsets (neutrophils, monocytes, and lymphocytes) are expressed as mean ± standard deviation from groups of five animals/evaluation time. Significant differences (p<0.05) between groups are represented by the letters a, b, c, and d, referring to the C, SAP, IFA, and MPL groups, respectively.

The results showed that there was initially a reduction in the number of total leukocytes in both the SAP and MPL groups in relation to the control groups. We also observed this reduction in a comparison of the SAP and IFA groups. These values increased after 24 h when compared to the IFA and MPL groups and, the high levels were maintained at 48, 96, and 336 h in the SAP group in relation to the MPL group or when compared to the control at the last time point (336 h). In the IFA group there was an increase in this parameter from 96 h in the MPL groups and in comparison to the control at 168 and 336 h. In blood cell subsets, we observed that neutrophils were increased in the SAP group at 12 h in relation to the control and IFA groups and at 24 and 48 h compared to other groups after inoculation. In the IFA group, there was an increase in the number of neutrophils at 168 h compared to the control. For the MPL group, neutrophils levels were enhanced at 12 h only in comparison with the control group. In addition, in the SAP group, the number of monocytes was increased at 48 in relation to the MPL group and at 336 h compared to the control, while in the IFA group there was an increase at 168 h compared to the control and at 336 h in relation to the control and MPL groups. Adjustments were also seen in the lymphocyte population with initial reductions in the SAP and MPL groups compared with the control and IFA groups. Furthermore, we detected differences in the lymphocyte populations between SAP and MPL groups. In the SAP group, this parameter was augmented at 96 h in relation to the MPL group and at 336 h compared to the control group. On the other hand, there was a reduction at 12 h in relation to the control and an increase at 96 h compared to the MPL group, and at 168 and 336 h in the IFA group in relation to control. Our results show that there were no changes induced by vaccine adjuvants in relation to number of red blood cells (data not shown).

**Figure 5:**
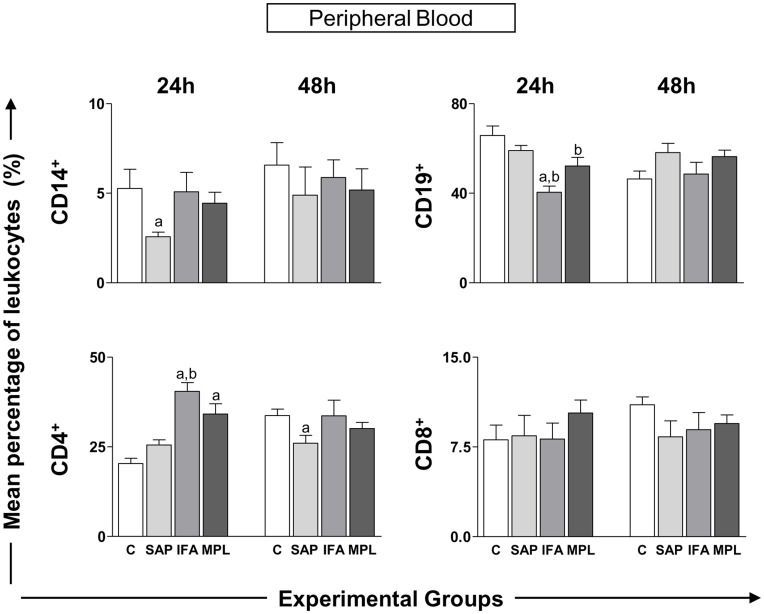
Leukocyte immunophenotypic profile in peripheral blood of mice sensitized with vaccine adjuvants: saponin (SAP; light gray), incomplete Freund’s adjuvant (IFA; medium gray), and monophosphoryl lipid A (MPL; dark gray) at 24 or 48 h after stimulation. The control group was inoculated with 0.9% sterile saline (C; white). The bar graphs present the percentage of cells expressing CD14^+^ (monocytes), CD19^+^ (B lymphocytes), and CD4^+^ and CD8^+^ (T-lymphocyte subsets). Significant differences (p<0.05) between groups are represented by the letters a, b, c and d, referring to the C, SAP, IFA, and MPL groups, respectively, and ANOVA following Tukey’s test was employed. Data presented are the mean±SD from groups of five animals/evaluation time.

In order to expand the investigation to subsets of circulating leukocytes, we carried out immunophenotyping to detect CD14^+^ monocytes, CD19^+^ B lymphocytes, and CD4^+^ or CD8^+^ T lymphocytes at two different times (24 and 48 h) after sensitization with vaccine adjuvants (Figure 5). At 24 h, there was a reduction of monocytes in the SAP group (2.57±0.78) in relation to control group. CD19^+^ lymphocytes at 24 h were reduced in the IFA group (40.44±8.10) in relation to the control and SAP groups and in the MPL group (52.13±11.74) compared to the control. With regard to the CD4^+^ lymphocytes at 24 h, we observed an increase in the IFA group (40.49±7.16) compared to the control and SAP groups, and in the MPL group (34.13±8.20) in relation to control group. Within 48 h, there was a reduction of this cell type in the SAP group (26.03±6.47) when compared to the control group. No differences were found in the CD8^+^ T-lymphocyte subset.

## Discussion

An effective early innate immune response to vaccine adjuvants may significantly impact the overall immunogenicity and efficacy of vaccines [15]. Vaccine adjuvants cause enhanced immunogenicity by increasing local inflammation, stimulating the proliferation of nonspecific lymphocytes, and prolonging the persistence of the antigen [16]. In this context, adjuvants play an important role since they enhance recruitment of various cells types of the innate immune system and the rate of antigen uptake and induce increased expression of endogenous inflammatory cytokines [17], [18]. However, little is known about the mechanisms involved in such cell recruitment [19], [20]. Our study present provides insight for both innate and adaptive immune responses induced by the vaccine adjuvants SAP, IFA, and MPL at 1, 12, 24, 48, 96, 168, and 336 h in mice sensitized with a single dose in the back.

The use of intradermal immunization was selected due of immunological properties that make the skin an attractive organ for the vaccine delivery. Moreover, the skin is the largest and most accessible organ and acts as a physical and immune barrier [21], [22]. The specific immunologic environment of the skin, known as the skin-associated lymphoid tissue, consists mainly of Langerhans cells and dermal antigen-presenting cells, which circulate between the skin and the lymph nodes. It also includes keratinocytes and endothelial cells, which produce a wide range of cytokines, and lymphocytes with passage from the circulation into the skin [23]–[25].

The quantification of the cellular infiltrate showed that all the adjuvants evaluated cause infiltration of immune cells into the injection site. Overall, our results showed that SAP, IFA, and MPL induce a rapid and local recruitment of inflammatory cells to the site of injection and this response is maintained up to 336 h after stimulation. It is important to note that this is a property shared by most of the commonly recognized adjuvants, and our data are consistent with results from other authors who have described the adjuvants’ ability to induce a local inflammatory reaction at an injection site [6], [26], [27]. Despite the local inflammatory reaction observed by optical microscopy, no macroscopical ulcerations were observed (data not shown), as previously documented by others [28], [29]. It has been suggested that the immune response is proportionally related to the tissue damage evoked by the adjuvants. New adjuvants may mimic danger signals but preferably with minimal injury to healthy tissue [26]. The presence of pro-inflammatory and regulatory cytokines is necessary for a balanced immune response and for the development of safe and more efficient vaccines [30]. In order to verify possible associations between the cytokines evaluated, correlation analyses were performed. We found an interesting strong positive correlation between IL-10 with IL-17 for all adjuvants evaluated (SAP: r = 0.8368, p<0.0001; IFA: r = 0.6687, p<0.0001; MPL: r = 0.7029, p<0.0001). These findings reinforce the importance to induce a balanced immunity with proinflammatory/regulatory profile by the adjuvants avoiding large injury to the tissues.

The beginning of inflammation can occur through the activation of pattern-recognition receptors, which are expressed in different forms and compositions by a variety of cells, including lymphocytes, granulocytes, and endothelial cells [31], [32]. The study of the cellular profile is very important, since it may be an important indicator of the local inflammatory process and directly involve the cytokine profile present in this microenvironment [33]. From this perspective, another important question raised in our study was the ability of vaccine adjuvants to promote differential recruitment of inflammatory cells to the inoculation site as well as activation of these cells.

Our data show differential migration of neutrophils to the injection site, and the presence of these cells was more persistent in the group sensitized with SAP at different time points. However, there was a peak of neutrophil migration at 12 h in all groups as compared to the control. Calabro *et al.* [6] in their studies with the MF59 adjuvant hypothesized that the action of this adjuvant is based on a central element of recruitment of innate immune cells to the injection site with successive waves of infiltrating cell populations, with neutrophils being the first and most abundant cell type to accumulate in the muscle, followed by inflammatory monocytes, eosinophils, and dendritic cells. These authors suggested that adjuvanticity of MF59 does not depend exclusively on neutrophils, pointing to a high degree of redundancy in the innate immune system or alternatively to other cell types that may be indispensable for the adjuvant effect of MF59. Besides, Smith *et al.* [34] observed intense local inflammation, with early recruitment of neutrophils and mast cells followed by macrophages, dendritic cells, and lymphocytes after administration with ISCOMS using intraperitoneal injection. Neutrophils are worth mentioning since they can induce subsequent selective waves of immune cell recruitment through secretion of chemokines. Alternatively or in addition, neutrophils may play an important role as the vehicle for transport vaccine antigen into the draining lymph nodes for further processing and presentation, presumably by dendritic cells [35]. Studies conducted by Yang *et al.* [36] showed a negative role of neutrophils in the adaptive response by CD4^+^ T lymphocytes and B lymphocytes after immunization with the antigen and different adjuvants. This work showed that neutrophils migrate rapidly from the lymph nodes draining the site of immunization through the lymphatic vessels; these cells can compete with antigen-presenting cells (dendritic cells and macrophages) in the antigen capture to presenting lymphocytes.

In the present study, we observed that macrophage and lymphocyte cell populations were also present at the inoculation site. Another possible target cell type for adjuvants is the macrophages that act as the first line of the immune system defense. They are located in tissues throughout the body, where they sense danger via a variety of molecules receptors, including LPS, mannose, CpG dinucleotides, and lipotechoic acid, that are conserved on pathogens through evolution [17]. The macrophages secrete a variety of cytokines, including IL-1, IL-6, IL-8, and TNF-α that mobilize innate immune reactions, and signal T cells via IL-l0, IL-12, and IL-18 to initiate specific responses against intracellular and extracellular pathogens [23]. The majority of dermal leukocytes are macrophages, which extravasate across the dermal venular walls and are well-differentiated. Macrophages scavenge the dermal microenvironment along the dermal side of the basement membrane to clear away antigen and participate in effector mechanisms at the site of injury [37].

Korsholm *et al*. [38] emphasized that adjuvants affect different cell populations at the injection site, leading to a rapid selective cellular recruitment. The mechanism of action of vaccine adjuvants must be addressed *in vivo* where different cell types cooperate in establishing an integrated immunocompetent environment [39].

Although the adjuvants (SAP, IFA, and MPL) are able to direct the immune response, each has a system that can differentiate the intensity or duration of the required immune response. Thus, after observing the ability of vaccine adjuvants in inducing inflammation and differential cell migration, we decided to evaluate the cytokine profile in order to better understand the early events related to precise immune response to adjuvants.

Our results show that SAP had a more subtly cytokine production with mixed immune profile with production of IL-2 as well as IL-6 and IL-4 after inoculation. Kensil *et al.* [40] demonstrated in mice, saponin is a potent adjuvant for CTL induction and promotes Th1 cytokine secretion (IL-2 and IFN-γ) and production of IgG2a. Behboudi *et al.* [41] observed that the ability of various *Quillaja* saponins in ISCOMATRIX formulations to induce pro-inflammatory cytokines, such as IL-1α and IL-6, and stimulate the acquired immune responses to influenza virus envelope proteins. The mechanism of saponins actions is not fully elucidated, but *in vitro* experiments suggest that saponins could improve antigen presentation by antigen-presenting cells and therefore optimize T cell response. Moreover, saponins have been demonstrated to improve B-cell response, although it remains to be established if this is through a direct effect or via antigen-presenting cell or T-cell stimulation [42].

In our work, IFA was also capable of inducing the production of type 1 cytokines, such as IFN-γ and TNF-α, at the final time points, and the analysis of the Th17 cytokine profile showed an increase in IL-6 and IL-17 at some later points. Moreover, we observed production of regulatory cytokines (IL-4 and IL-10) at 24 and 48 h. It is already known that the combination of paraffin oil and surfactant as components of IFA, might exert various regional and/or systemic effects on the immune system [43]. However, little information is available on cytokine induction by IFA when it is administered alone. In rats receiving IFA, there was a rapid increase in the mRNA for TNF-α, with limited IFN-γ mRNA expression, and no mRNA expression for IL-2 was observed. Remarkably, when ovalbumin was combined with IFA, the expression of IL-4 mRNA rather than TNF-α mRNA was detected [44], suggesting a shift toward Th2 responsiveness.

Our results showed that among the evaluated vaccine adjuvants, MPL was able to induce the highest production of type 1 cytokines (IL-2, TNF-α, and IFN-γ) at different time points as compared to the control, SAP and, IFA groups. This adjuvant was also an important inducer of type 17 cytokines (IL-6 and IL-17). In the present study, MPL also showed increased levels of IL-10, and these results might be related to this adjuvant being an important factor in TLR4 stimulation on activation of the innate immune response, by activating NF-κB transcriptional activity and the subsequent expression of pro-inflammatory cytokines, such as TNF-α and IL-6. Pro-inflammatory cytokines, secreted by resident and recruited cells, directly stimulate cells that then present the antigen in the draining lymph node [45]. The contribution of MPL in the innate immune response at the injection site was demonstrated by further analysis of the proinflammatory cytokine (IL-6, TNF-α, and IFN-γ) and chemokine (CCL2/MCP-1 and CCL3/MIP-1α) levels in homogenates prepared from injected muscle at 3, 6, and 24 h and 7 days post-injection [27]. In this sense we agree with Morel *et al*. [46] who used AS03 and observed a similar heterogeneity in cytokine kinetics, which probably reflected differences in the regulation of their expression and/or in the activity of different waves of recruited cells.

Herein, we also evaluated the systemic response through the hematological fluctuation of the total leukocytes and their subsets, mainly neutrophils, monocytes, and lymphocytes. After sensitization, even at early events occurring in the first moments of the response, a reduced number of total leukocytes were observed in the SAP, IFA, and MPL groups. Nevertheless we observed increased leukocyte values in the SAP and IFA groups at the later times points. These data suggested that leukocytes initially migrate to the focus of the stimuli as demonstrated by the reduction of these cells in circulating peripheral blood. Regarding leukocyte subsets, we detected increased neutrophils counts in the SAP group at 12, 24, and 48 h after inoculation. In contrast, we observed an increase in the number of neutrophils at 168 and 336 h in the IFA group. These increases were also present in the MPL group, but at 12 h only. These findings are congruent with neutrophils being able to quickly mobilize from the bone marrow to the blood in response to infections or immunizations [47], [48], and these findings can be elucidated by the early increase of neutrophils in the SAP and MPL groups. The analysis of monocytes showed changes at some time points in the SAP group, specifically increased monocytes at 48 and 336 h. On the other hand, there was an increase of monocytes at 168 and 336 h in the IFA group. Changes were also seen in the lymphocyte population with initial reductions in the SAP and MPL groups. In relation to lymphocytes population, we can observe a consistent pattern, with the early phase of the immune response characterized by a sharp decline in the lymphocytes in the lymph nodes and subsequent stimulated recruitment of efferent blood lymphocytes [49]. However, lymphocytes continuously move, via the blood stream and lymphatics, from one peripheral organ to another and to inflammation sites [25]. In the SAP group, these values were elevated at 96 and 336 h. In the IFA group, a reduction at 12 h and an increase at 96, 168, and 336 h were observed. Interestingly, we observed a delayed expansion of neutrophils, monocytes, and lymphocytes in the animals sensitized with IFA. This may reflect a persistence of the emulsion at the site of injection (oil droplets) and an ability to stimulate innate immune cells later [50].

We also evaluated the percentage of circulating CD14^+^ monocytes, T lymphocytes subsets (CD4^+^ and CD8^+^), and B lymphocytes by flow cytometry. Considering the results found in the monocytes we observed a significant reduction at 24 hours in the SAP group; a reduction in the percentage of CD19^+^ lymphocytes (B lymphocytes) in both the IFA and MPL groups was also detected. In contrast, we observed an increase in the percentage of CD4^+^ T-lymphocytes in these same groups. Our data suggest that a vaccine adjuvant causes adjustment at the site of inoculation, probably inducing a response in the draining lymph node, resulting in changes in leukocyte influx. The sensitization of the skin with vaccine adjuvants caused small changes in this influx and the profile of some circulating cells. These alterations were most prominent in SAP and IFA groups, but more subtle in the MPL group. These data are important since different authors have demonstrated relevant results about the local innate responses focused at the injection site can be related to the development of minimum vaccine risks [27], [46].

Overall, our data suggest that the evaluated vaccine adjuvants contribute to the cell recruitment, with different cell types culminating in distinct cytokine profiles. This event is important in the establishing and integration of the immunocompetent environment, favorable for antigen processing, presentation, and subsequent cellular immune response stimulation. However, further studies are still needed to evaluate and identify which cell types are essential to induce the appropriate response. Even so, our data reinforce the importance of activating the innate immune response to establish a robust, specific immune response after an immunization.
